# Postoperative Morbidity and Mortality of Perforated Peptic Ulcer: Retrospective Cohort Study of Risk Factors among Black Africans in Côte d'Ivoire

**DOI:** 10.1155/2016/2640730

**Published:** 2016-01-26

**Authors:** Soro Kountele Gona, Mahassadi Kouamé Alassan, Koffi Gnangoran Marcellin, Kissi Ya Henriette, Coulibaly Adama, Assohoun Toussaint, Ehua Adjoba Manuela, Seu Gagon Sylvain, Afum-Adjei Awuah Anthony, Ehua Somian Francis

**Affiliations:** ^1^Service de Chirurgie Digestive et Viscérale, CHU de Yopougon, Côte d'Ivoire; ^2^Service de Médecine et d'Hépato-Gastroentérologie, CHU de Yopougon, Côte d'Ivoire; ^3^Kumasi Centre of Collaborative Research in Tropical Medicine, Ghana; ^4^Service des Urgences Médico-Chirurgicales, CHU de Yopougon, Côte d'Ivoire

## Abstract

*Introduction*. Surgical treatment of perforated peptic ulcer (PPU) is a challenge for surgeons in Africa.* Aim*. To determine risk factors of postoperative complications or mortality among black Ivoirian patients with PPU.* Methods*. All 161 patients (median age = 34 years, 90.7 male) operated on for PPU in the visceral and general surgery unit were enrolled in a retrospective cohort study. Variables were studied with Kaplan Meier and Cox proportional hazard models.* Results*. Among 161 patients operated on for PPU, 36 (27.5%) experienced complications and 31 (19.3%) died. Follow-up results were the incidence of complications and mortality of 6.4 (95% CI: 4.9–8.0) per 100 person-days and 3.0 (95% CI: 1.9–4.0) per 100 person-days for incidence of mortality. In multivariate analysis, risk factors of postoperative complications or mortality were comorbidities (HR = 2.1, *P* = 0.03), tachycardia (pulse rate > 100/minutes) (HR = 2.4, *P* = 0.02), purulent intra-abdominal fluid collection (HR = 2.1, *P* = 0.04), hyponatremia (median value ≤ 134 mEq/L) (HR = 2.3, *P* = 0.01), delayed time of hospital admission > 72 hours (HR = 2.6, *P* < 0.0001), and delayed time of surgical intervention between 24 and 48 hours (HR = 3.8, *P* < 0.0001).* Conclusion*. The delayed hospital admission or surgical intervention and hyponatremia may be considered as additional risk of postoperative complications or mortality in Black African patients with PPU.

## 1. Introduction

Perforated peptic ulcer (PPU) is one of the most deadly gastrointestinal complications accounting for approximately 23.5% of mortality in Europe [1]. Many studies have been focused on risk factors of morbidity and mortality of PPU with debating results mainly in western and Asian countries. These studies report that age, abdominal sepsis, comorbidities, and delayed treatment are risk factors of mortality in patients with PPU [2–6].

In Africa, despite the introduction of new drugs and recommended guidelines, treatment of peptic ulcer remains a challenge in most African countries, because of the high cost of medications and cultural behaviors [7–10]. Therefore, many patients with peptic ulcer are seen in critical conditions as the results of complications such as perforation, hemorrhage, or gastric outlet obstruction [7, 11].

PPU accounted for 4.6 to 29.7% of emergency surgery in Africa with in-hospital mortality rate reaching 11% [12–14]. Many risk factors of mortality and morbidity have been studied with conflicting results related to varying methodology [12–16].

Therefore data on factors affecting the outcome of patients with PPU are scanty in Africa as in Côte d'Ivoire. The knowledge of these factors that affected the outcome would allow surgeons in Côte d'Ivoire to prevent postoperative complications in patients with PPU.

This study is aimed at providing predictive factors of morbidity and mortality in black Africans with PPU at the Yopougon teaching hospital in Abidjan.

## 2. Methods

### 2.1. Study Site

This study was conducted at the Yopougon teaching hospital. Yopougon is the widest suburb located at the north of Abidjan, the economic capital of Côte d'Ivoire. The teaching hospital has more than 300 beds and was inaugurated in 1996. It has a general and visceral surgery unit dedicated to adult patients and a surgical emergency unit.

### 2.2. Patients

We conducted a retrospective cohort study among PPU patients undergoing surgery in a periurban setting in Abidjan, Côte d'Ivoire.

Patients admitted for acute abdominal conditions at the surgical emergency unit of the Yopougon teaching hospital, from January 2000 to December 2013, were enrolled in this study. Patients included were those operated on for PPU and hospitalized in the general and visceral surgery unit. Those who aged less than 15 years, who were operated on previously for PPU, and having hemorrhagic peptic ulcer, gastrointestinal stenosis, or gastrointestinal perforation from other origins (caustic, cancer, foreign bodies, and typhoid) were excluded.

### 2.3. Patient's Admission and Intervention

Patients with acute abdominal symptoms were admitted to emergency at the Yopougon Teaching Hospital. Those with suspected acute peritonitis [17, 18] underwent plain X-ray of abdomen in standing and laying position. The presence of free gas under the dome of diaphragm is suggestive of perforation of digestive tract organ [18]. Perfusion with saline solution and systematic antibiotic chemotherapy (cefotaxime 1 g BID and metronidazole 500 mg BID) intravenously were immediately administered and patients underwent biological examinations including full blood counts, creatinine, urea, blood glucose, blood group, natremia, kalemia, and chest X ray. PPU was definitely ascertained when ulcer perforation was seen during surgery procedure [18]. Treatment of perforation was mainly excision and suture of the hole with omentoplasty after abdominal lavage with 5% of saline solution and antiseptic [18, 19]. Only one patient had had partial gastrectomy. None of them have had vagotomy [19].

Remaining fluid in abdominal cavity was evacuated by the drains inserted in the abdomen flanks. After surgical intervention, patients with immediate postoperative complications (i.e., choc, heart failure) were admitted in the intensive care unit at the same hospital. Patients who recovered and those without immediate complications were admitted in the GVSU for nursing. All patients were discharged after one week, more or less, unless complications or death occurred during hospitalization. Patients were seen as outpatients one week after discharge for clinic examination.

### 2.4. Predictor Variables

For all patients included, sociodemographics, clinic variables, biologic variables at admission, and pre- and postoperative variables were retrieved.

Variables retrieved at admission were age, sex, occupation, presumable date of acute symptom's onset, date of admission at emergency unit, medical history of peptic ulcer, history of anti-inflammatory drugs, alcohol intake, smoking, presence and type of comorbidities, blood pressure, hemoglobin, hematocrit, platelets count, natremia, kalemia, prothrombin time, and clinical records comprising pulse rate, blood pressure, clinical signs of peritonitis, and the American score of anesthesiologists (ASA) [20]. Pre- and postoperative variables were date of surgical intervention, surgeon skills (junior or senior), type of surgical intervention and duration, perforation site (stomach or duodenal), quality of intra-abdominal fluid (pus or bile-like fluid), and type of postoperative complications [21].

### 2.5. Definitions

PPU was diagnosed with the combination of clinical symptoms of peritonitis and radiological signs of digestive tract perforations and ascertained after laparotomy [7, 18, 19].

Complications were defined as any life-threatening conditions occurring before, during, or after surgery necessitating resuscitation measures or medical or surgical treatment [21].

Mortality was defined as any death occurring during or after surgical intervention before hospital discharge.

Junior surgeons were residents in surgery unit with less than 5 years of experiences.

Senior surgeons were surgical staff members with surgery diploma and more than 5-year experience.

Treatment delay denotes time interval from acute symptoms onset to surgical intervention [22]. We split this variable into two variables as follows:

 Delay to emergency admission (named admission delay) denoted time interval from acute symptoms onset to emergency unit admission which also means duration of symptoms.
 Delay to surgical intervention (named intervention delay) denoted time interval from emergency admission to surgical intervention.
 Early treatment delay or hospital admission denoted time delay less than 24 hours.

We used these definitions because preoperative work-up depends on the capability and swiftness with which patients or their family face medical expenditure as no social security existed in Côte d'Ivoire. In conjunction with cultural belief, these factors may delay surgical treatment [10, 16].

Hyponatremia is defined by a level of natremia ≤134 mEq/L [23].

Tachycardia is defined as cardiac pulse rate more than 100 beats/minute.

### 2.6. Statistical Analysis

The main outcome was the occurrence of postoperative complications or death during hospitalization as dependant variable and the second outcome was the occurrence of death. Categorical variables were expressed as numbers and percentages and continuous variables as median, range, or mean and standard deviation if appropriated. Continuous variables were dichotomized using median value as cut-off if appropriated. Complications or death was used as time dependant variables because the date of their occurrences varied between patients during follow-up and allowed us to determine the person-time at risk [24, 25]. The time of origin was the date of surgical intervention. Patients were censored if death or complications did not occur before the discharge. The person-time at risk was the sum of individual number of days of observation during follow-up. The incidence of complications or mortality was determined as number of complications or death divided by person-time at risk multiplied by 100 and expressed as number of complications or mortality per 100 person-days [24]. The survival probability (no occurrence of death or complications) was expressed by Kaplan Meier curve. Univariate and multivariate Cox regression analyses were used to compare variables [25]. Level of significance was set at 0.05. All statistical analyses were computed with SPSS version 16.0 (SPSS Inc., Chicago, IL, USA) and SAS version 9.2 (SAS Inc., Cary, NC, USA) software.

## 3. Results

### 3.1. Description of Enrolled Patients

Demographic, clinic, and biologic characteristics of enrolled patients were depicted in Table 1. Patients with PPU were younger (median age 34 years), mostly men (90.7%), and 45.3% of them were admitted in emergency unit within 24 hours after the onset of symptoms; 73.9% had comorbidities upon admission. The main comorbidities were weight loss (57.1%), arterial hypertension (4.3%), diabetes (2.5%), diabetes and arterial hypertension (4.3%), renal insufficiency (1.8%), and heart disease (3.1%). Patients were mostly operated on by senior surgeons (64.6%) with delayed surgical intervention up to 24 hours in 85.7% of cases. After laparotomy, the site of perforation was located mostly in the duodenum (53.4%) compared to the antrum (46.6%) and purulent intra-abdominal fluid collection was seen in 39 (24.2%) patients. The median duration of surgical intervention was 76 minutes. The main operation procedure performed was simple closure of the perforation. Partial gastrectomy with Billroth 2 procedure was done in one patient.

### 3.2. Morbidity and Mortality

**Figure 1:**
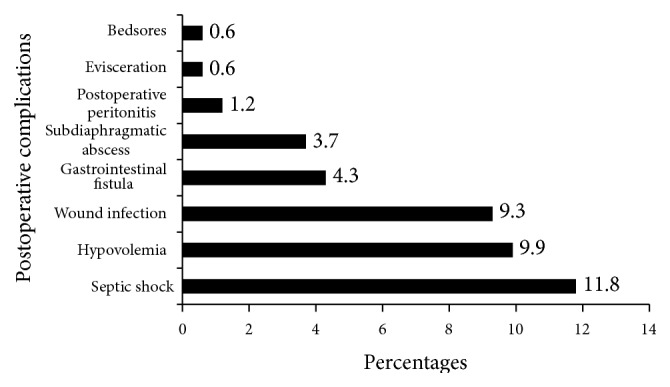
Frequencies and type of postoperative complications.

Among 161 patients operated on for PPU, 36 (27.5%) experienced complications and 31 (19.3%) died. All were followed up for 1042 person-days resulting in incidence rate of complications and mortality of 6.4 (95% CI: 4.9–8.0) per 100 person-days and 3.0 (95% CI: 1.9–4.0) per 100 person-days for mortality rate. Patients with postoperative complications or death had high median value of white blood cells (*P* < 0.0001), high cardiac pulsation (*P* < 0.0001), low level of natremia (134 versus 137, *P* = 0.02), and kalemia (3.6 versus 3.7, *P* = 0.01) compared to those without (Table 1). The most frequent postoperative complications were septic shock (11.8%), hypovolaemia (9.9%), wound sepsis (9.3%), and gastrointestinal fistula (4.3%) (Figure 1). Septic shock occurred more frequently in deceased patients (61.3% versus 0.0%, *P* < 0.0001) compared to those with only complications.

### 3.3. Risk Factors of Morbidity and Mortality

**Figure 2:**
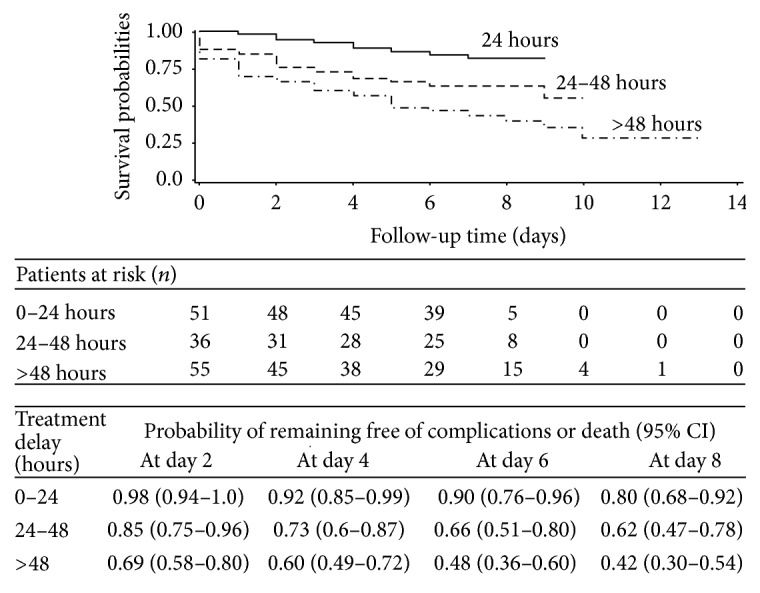
Kaplan Meier curves of probability of remaining free of postoperative complications or death according to the delay of treatment and length of hospital stay since surgical intervention.

**Figure 3:**
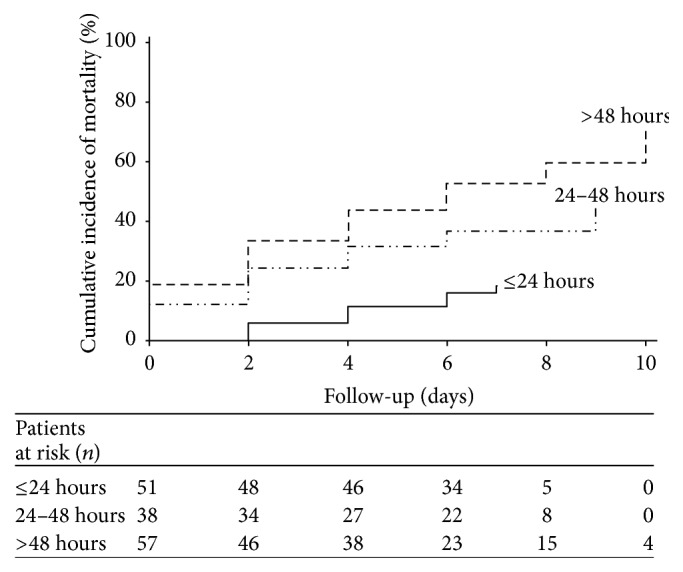
Cumulative incidence of postoperative mortality according to the delay of treatment.

The median survival time free of postoperative complications or death was 16 days (95% CI: 9–18) which increased with the absence of comorbidities (Log rank test = 5.8, *P* = 0.02), the absence of purulent intra-abdominal fluid collection (Log rank test = 44.4, *P* = 0.0001), and short delay of surgical intervention less than 24 hours after hospital admission (log rank test = 13.14, *P* = 0.003). Overall, patients operated on early (treatment delay less than 24 hours) had better outcome (log rank test = 21.03, *P* < 0.0001) (Figure 2) with low cumulative incidence rate of postoperative mortality compared to those with treatment delay up to 24 hours (Figure 3) during follow-up. In multivariate analysis risk factors of postoperative complications and death were comorbidities (HR = 2.1, *P* = 0.03), tachycardia (pulse > 100/minutes) (HR = 2.4; *P* = 0.02), purulent intra-abdominal fluid collection (HR = 2.1, *P* = 0.04), hyponatremia (≤134 mEq/L) (HR = 2.3, *P* = 0.01), delayed hospital admission > 72 hours (HR = 2.6; *P* < 0.0001), and delayed surgical intervention between 24 and 48 hours (HR = 3.8, *P* < 0.0001). Moreover, using mortality as outcome, only natremia and tachycardia were not risky and the other factors remained at risk in multivariable analysis (Table 2).

## 4. Discussion

We demonstrated in this study that the postoperative incidence of morbidity and mortality was high in Black Africans operated on for PPU. This incidence was related to comorbidities, tachycardia, hyponatremia, purulent abdominal fluid, and long treatment delay such as a delay of hospital admission and delayed surgical intervention since hospital admission.

Comorbidities related to heart or pulmonary diseases, renal insufficiency, or diabetes are risk factors of mortality [1–5]. In addition to these life-threatening comorbidities, weight loss was the major comorbidity reported among our patients probably due to the avoidance of food and metabolic disorder related to peptic ulcer disease [7, 26].

It is obvious that the purulent intra-abdominal fluid collection is risk factor of complications or death and may lead to septic shock as experienced by 11.8% of patients in our study probably due to the long lasting perforation before the administration of appropriate treatment as stated elsewhere in Africa [1, 14, 16]. Septic shock raises mortality rate up to 50% in patients with PPU, as in our study, whose main symptom tachycardia is also known to be a risk factor of mortality [1, 5, 27]. However tachycardia may reflect heart disease or arterial hypertension presented by 7.4% of patients in this study. However any correlation found between these comorbidities and tachycardia (*P* = 0.14) indicates a bias effect [28].

Hyponatremia is common in patients with perforated peritonitis regardless of the segment of digestive tract involved and related to third spacing of gastrointestinal fluid leakage into the abdominal cavity [23, 29]. Previous studies have shown that hyponatremia predicts poor outcome in case of spontaneous bacterial peritonitis or peritoneal dialysis related peritonitis [30, 31]. But its influence on the outcome of Black African patients with PPU peritonitis is not clearly demonstrated. Our study showed that hyponatremia increased by twofold the risk of complication or death in Black Africans with PPU. This finding emphasized the need for intensive resuscitation of these patients before surgical treatment. In fact, the level of natremia at baseline was significantly low in patients with complications and death compared to those without. However, hyponatremia failed to predict mortality which is apparently related to the low sample size illustrated by wide confidence intervals around the point estimates of risk factors for this outcome [32].

Therefore, patients with PPU must be treated promptly when symptoms occur and diagnosis is done [7, 17]. In accordance with previous studies, we demonstrated that delayed time to surgical treatment was one of the key factors leading to complications or death [12, 14, 22]. Possible reasons for this observation were that, in Africa, patients usually lengthened the waiting time before hospital admission, which implied long duration of symptoms because they often use herbal medicine according to their cultural beliefs or receive symptomatic treatment in primary care units to relieve pain [10, 13, 15]. Furthermore, in our hospital, medical fees are not free of charge and most patients have low income, as stated elsewhere, in Africa [12] and could not afford medical expenditure that allows them to be promptly treated as 85% of patients were operated on more than 24 hours after hospital admission in our study.

This study has some limitations. One is related to retrospective study subject to bias [28, 33]. Firstly, the nonsignificant effect of ASA score may be related to information bias as only three levels among five of ASA score were depicted although this determination is subjective [34]. Secondly the main outcome was composite and did not allow us to use common prognostic scores in PPU that almost measured the 30-day mortality [20, 34, 35]. But we think that composite variable combining complications and death was relevant in our clinical practice shadowed by limited medical facilities as 22.9% of complications (hypovolaemia, septic shock, and postoperative peritonitis) were life-threatening conditions [21].

However, this study was strengthened by the epidemiological profile of patients with PPU who were younger resulting in nonsignificant effect of age. This profile is previously reported in other countries in Africa [12, 13]. Almost all of them underwent the same surgical procedure, except one gastrectomy, minimizing the confounding effect of surgical procedure on the outcome [28]. Moreover, our methodological approach demonstrated the magnitude of postoperative complications and risk factors involved, through timely recorded variables from African patients with PPU [24, 25]. Finally risk factors of morbidity and mortality found in this study are those commonly reported in previous studies except for age and ASA score.

## 5. Conclusion

In Côte d'Ivoire the delayed hospital admission or surgical intervention and hyponatremia may be considered as additional risk that may work in concomitance with previously documented known risk factors of postoperative complications or mortality in patients with PPU. These findings could be used as a guide by surgeons to monitor African patients with PPU for a better outcome after surgical intervention in hospital with limited medical facilities.

## Figures and Tables

**Table 1:** Demographic, clinic, and biologic variables.

Parameters	All patients *N* = 161	Complications or death		
		No	Yes	*P*
		*n* = 94	*n* = 67	
Age (years) [median (range)]	34 (72)	33.5 (54)	35 (69)	0.17
Sex (male) [*n* (%)]	146 (90.7)	88 (93.6)	58 (86.6)	0.2
Pulse (pulsations/minute) [median (range)]	98 (72)	95 (50)	110 (72)	0.0001
Hemoglobin (g/dL) [median (range)]	13.8 (9)	14 (5)	13.6 (9)	0.13
Hematocrit (%) [median (range)]	41 (23)	41 (7)	41 (23)	0.64
WBC (10^2^/mL) [median (range)]	110 (320)	107 (110)	130 (320)	0.0001
Platelets count (10^3^ cel/mL) [median (range)]	199 (493)	206 (492)	190 (425)	0.06
Prothrombin time (%) [median (range)]	89.5 (52)	90 (33)	88 (52)	0.18
Creatinine (g/dL) [median (range)]	17.8 (95)	13 (17)	19 (95)	0.02
Kalemia (Meq/mL) [median (range)]	3.7 (1.9)	3.7 (1.5)	3.6 (1.8)	0.01
Natremia (Meq/mL) [median (range)]	136 (33)	137 (27)	134 (33)	0.02
Arterial hypertension (yes) [*n* (%)]	9 (5.6)	6 (6.4)	3 (4.5)	0.73
Smoking (yes) [*n* (%)]	126 (78.3)	78 (83)	48 (71.6)	0.12
Anti-inflammatory drugs (yes) [*n* (%)]	59 (36.6)	34 (36.2)	25 (37.3)	1.00
Comorbidities (yes)	119 (73.9)	8 (8.5)	19 (28.4)	0.003
Surgeons skills (yes) [*n* (%)]				0.12
Junior	57 (35.4)	37 (39.4)	20 (29.9)	
Senior	104 (64.6)	57 (60.6)	47 (70.1)	
Intra-abdominal fluid collection [*n* (%)]				0.0001
Bile-like fluid	121 (75.2)	90 (95.7)	31 (46.3)	
Purulent	39 (24.2)	3 (3.2)	36 (53.7)	
Site of perforation [*n* (%)]				0.3
Antrum	75 (46.6)	40 (42.6)	35 (52.2)	
Duodenum	86 (53.4)	54 (57.4)	32 (47.8)	
ASA score [*n* (%)]				0.0001
ASA1	0 (0.0)	0 (0.0)	33 (49.3)	
ASA2	127 (78.9)	94 (100)	34 (50.7)	
ASA3	34 (21.1)	0 (0.0)	0 (0.0)	
ASA4	0 (0.0)	0 (0.0)	0 (0.0)	
ASA5	0 (0.0)	0 (0.0)	0 (0.0)	
Hospital admission delay (hours) [*n* (%)]				0.0001
≤24 hours	73 (45.3)	54 (57.5)	19 (28.4)	
24–48 hours	50 (31.1)	26 (27.7)	24 (35.8)	
48–72 hours	18 (11.2)	11 (11.7)	7 (10.5)	
>72 hours	20 (12.4)	3 (3.2)	17 (25.4)	
Surgical intervention delay (hours) [*n* (%)]				0.0001
≤24 hours	23 (14.3)	6 (6.4)	17 (25.4)	
>24 hours	138 (85.7)	88 (93.6)	50 (74.6)	
Duration of surgical intervention (minutes)	76 (97)	76 (68)	75 (97)	0.6
Duration of hospital stay (days)				0.0001
[mean (SD)]	9.2 (5)	8 (1.8)	11 (7.1)	
[median (range)]	8 (36)	8 (12)	11 (37)	
Complications [*n* (%)]	36 (22.4)	—	36 (22.4)	
Death [*n* (%)]	31 (19.3)	—	31 (19.3)	

SD: standard deviation.

**Table 2:** Risk factors of postoperative complications or mortality (A) or mortality (B). Cox regression multivariate analysis.

Risk factors	A		B	
	HR	95% CI	HR	95% CI
Comorbidities				
No	1		1	
Yes	2.1	1.1–3.9	3.9	1.5–10.3
Pulse				
<100	1			
≥100	2.4	1.1–4.9		
Intra-abdominal fluid collection				
Bile-like fluid	1		1	
Purulent	2.1	1.03–4.2	6.4	2.4–17.1
Natremia				
≥134	1			
<134	2.3	1.2–4.2		
Hospital admission delay (hours)				
≤24 hours	1		1	
24–48	1.02	0.5–2.3	0.5	0.1–2.4
48–72	0.9	0.3–2.4	1.9	0.4–8.0
>72	2.6	1.2–5.7	13.5	3.9–46.0
Surgical intervention delay (hours)				
≤24	1		1	
24–48	3.8	1.7–8.5	15.6	4.9–49.2
>48	1.3	0.4–4.1	1.7	0.3–9.3

HR: hazard ratio; CI: confidence interval.
